# Salvinorin B meth­oxy­methyl ether

**DOI:** 10.1107/S1600536812043449

**Published:** 2012-10-27

**Authors:** Thomas A. Munro, Douglas M. Ho, Bruce M. Cohen

**Affiliations:** a McLean Hospital, Belmont, MA, USA; b Harvard Medical School, Department of Psychiatry, Boston, MA, USA; c Harvard University, Department of Chemistry and Chemical Biology, Cambridge, MA, USA

## Abstract

The title compound [MOM-SalB; systematic name: methyl (2*S*,4a*R*,6a*R*,7*R*,9*S*,10a*S*,10b*R*)-2-(3-fur­yl)-9-meth­oxy­meth­oxy-6a,10b-dimethyl-4,10-dioxo-2,4a,5,6,7,8,9,10a-octa­hydro-1*H*-benzo[*f*]isochromene-7-carboxyl­ate], C_23_H_30_O_8_, is a deriv­ative of the κ-opioid salvinorin A with enhanced potency, selectivity, and duration of action. Superimposition of their crystal structures reveals, surprisingly, that the terminal C and O atoms of the MOM group overlap with the corresponding atoms in salvinorin A, which are separated by an additional bond. This counter-intuitive isosterism is possible because the MOM ether adopts the ‘classic anomeric’ conformation (*gauche*–*gauche*), tracing a helix around the planar acetate of salvinorin A. This overlap is not seen in the recently reported structure of the tetra­hydro­pyranyl ether, which is less potent. The classic anomeric conformation is strongly favoured in alk­oxy­methyl ethers, but not in substituted acetals, which may contribute to their reduced potency. This structure may prove useful in evaluating models of the activated κ-opioid receptor.

## Related literature

For preparation, see: Béguin *et al.* (2009 ▶). For amended characterization data, see: Munro *et al.* (2008 ▶). For structure–activity relationships *in vitro*, see: Béguin *et al.* (2012 ▶); Munro *et al.* (2008 ▶); Prevatt-Smith *et al.* (2011 ▶). For *in vivo* pharmacology, see: Baker *et al.* (2009 ▶); Peet & Baker (2011 ▶); Wang *et al.* (2008 ▶). For pharmacokinetics and PET imaging of the eth­oxy­methyl ether, see: Hooker *et al.* (2009 ▶). For structure–activity relationships of salvinorin A, see: Cunningham *et al.* (2011 ▶). For crystal structures of related compounds, see: Ortega *et al.* (1982 ▶); Prevatt-Smith *et al.* (2011 ▶); Tidgewell *et al.* (2006 ▶). For solid-state and bioactive conformations of acetals, see: Anderson (2000 ▶); Brameld *et al.* (2008 ▶).
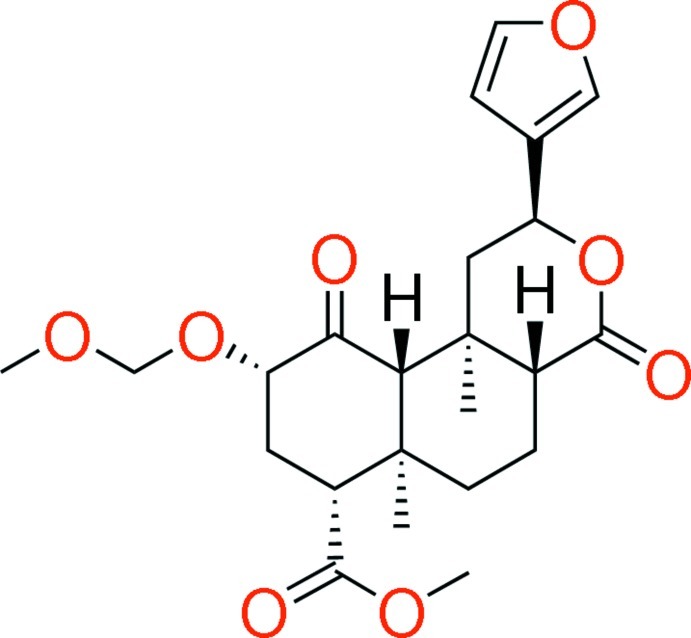


## Experimental

### 

#### Crystal data

C_23_H_30_O_8_
*M* *_r_* = 434.47
Monoclinic, 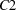

*a* = 27.8848 (7) Å
*b* = 6.2415 (2) Å
*c* = 12.8212 (3) Å
β = 107.351 (1)°
*V* = 2129.9 (1) Å^3^
*Z* = 4
Mo *K*α radiation
μ = 0.10 mm^−1^
*T* = 193 K
0.25 × 0.13 × 0.07 mm

#### Data collection

Bruker APEXII CCD diffractometer
Absorption correction: multi-scan (*SADABS*; Bruker, 2004 ▶) *T*_min_ = 0.975, *T*_max_ = 0.993
27784 measured reflections
6195 independent reflections
5296 reflections with *I* > 2σ(*I*)
*R*_int_ = 0.033

#### Refinement

*R*[*F* ^2^ > 2σ(*F* ^2^)] = 0.040
*wR*(*F* ^2^) = 0.108
*S* = 1.04
6195 reflections
330 parameters
181 restraints
H-atom parameters constrained
Δρ_max_ = 0.28 e Å^−3^
Δρ_min_ = −0.16 e Å^−3^

### 

Data collection: *APEX2* (Bruker, 2006 ▶); cell refinement: *SAINT* (Bruker, 2006 ▶); data reduction: *SAINT*; program(s) used to solve structure: *SHELXTL* (Sheldrick, 2008 ▶); program(s) used to refine structure: *SHELXTL*; molecular graphics: *ORTEP-3* (Farrugia, 1997 ▶) and *pyMOL* (DeLano, 2009 ▶); software used to prepare material for publication: *SHELXTL*.

## Supplementary Material

Crystal structure: contains datablock(s) global, I. DOI: 10.1107/S1600536812043449/qm2086sup1.cif

Structure factors: contains datablock(s) I. DOI: 10.1107/S1600536812043449/qm2086Isup2.hkl

Supplementary material file. DOI: 10.1107/S1600536812043449/qm2086Isup3.cdx

Supplementary material file. DOI: 10.1107/S1600536812043449/qm2086Isup4.cml

Additional supplementary materials:  crystallographic information; 3D view; checkCIF report

## Figures and Tables

**Fig. 1.**
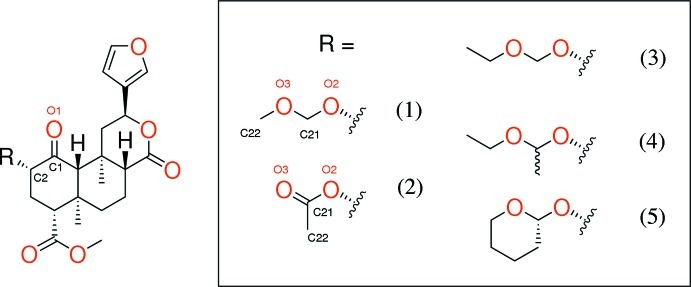
Structures of compounds discussed.

**Fig. 2.**
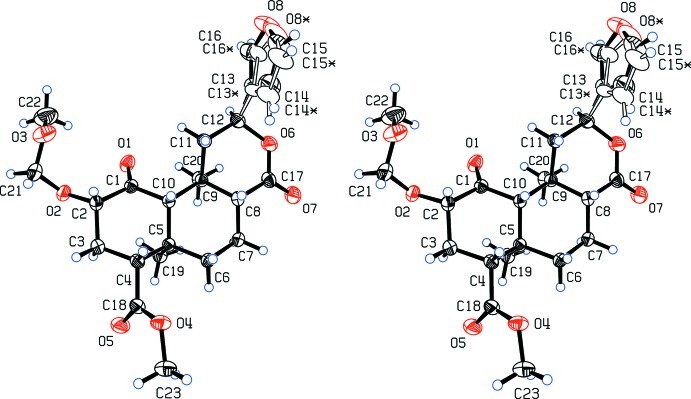
Crystal structure of (1) with 50% probability thermal displacement ellipsoids. Atom numbering follows the crystal structure of (2) (Ortega et al., 1982) (* = minor component of disorder) (Cross-eyed stereoview).

**Fig. 3.**
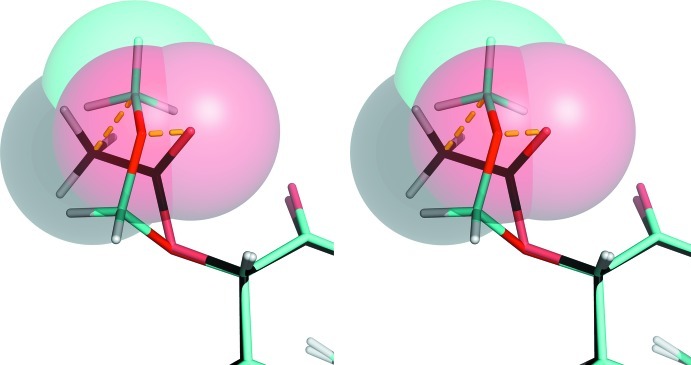
Superimposed structures of (1) in blue and (2) in black (CSD code BUJJIZ, Ortega et al., 1982). Spheres represent the van der Waals surfaces of O3 and C22, connected by dotted yellow lines (Cross-eyed stereoview).

**Fig. 4.**
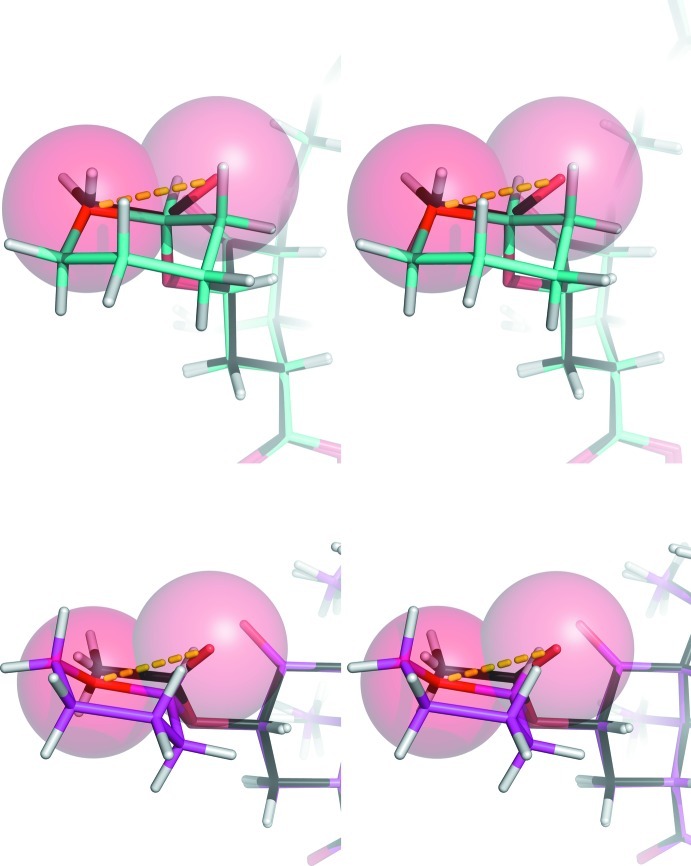
Superimposed structures of (2) in black with (5) in blue (major component) and purple (minor component)(CCDC 837303, Prevatt-Smith et al., 2011). Spheres represent the van der Waals surface of O3, connected by dotted yellow lines (Cross-eyed stereoview).

## supplementary crystallographic information

### Comment

Salvinorin B methoxymethyl ether (1) is among the most potent and selective κ (kappa) opioids known, with subnanomolar affinity and potency (Wang *et al.*, 2008). A semisynthetic derivative of the naturally occurring κ opioid salvinorin A (2), (1) was the first derivative reported to be more potent than (2) *in vitro*, and also showed greater potency and duration of action in mice (Wang *et al.*, 2008). The extreme potency of (1) has been confirmed both *in vitro* (Munro *et al.*, 2008, Prevatt-Smith *et al.*, 2011) and in rats (Baker *et al.*, 2009, Peet & Baker, 2011). The name MOM-SalB is widely used; the incorrect name `2-methoxymethylsalvinorin B', implying that the substituent is directly attached to C2, should be avoided.

In Figure 1, the structures of (1) and (2) have been drawn to emphasize their similarity, with O2, C21 and O3 superimposable. The terminal methyl group C22 is attached to O3 in (1) but C21 in (2), and might be expected to interact with different regions of the receptor. Extensive research has been done into the structure-activity relationships of (2), especially the role of the C2 acetate. The deacetyl analogue salvinorin B is at least 60-fold less potent than (2). Deoxygenation or demethylation of the acetate causes smaller reductions in potency (Cunningham *et al.*, 2011). This suggests that the two extremities of the acetate (O3 and C22) engage in separate, synergistic interactions with the binding pocket (Munro *et al.*, 2008). The structure-activity relationships of (1) have also been explored. Potency is dramatically reduced by replacement of O3 with sulfur or carbon (Munro *et al.*, 2008); this similarity to (2) is consistent with the proposed common binding pose. The ethoxymethyl ether (3) appears to be even more potent and selective than (1), both *in vitro* (Munro *et al.*, 2008, Prevatt-Smith *et al.*, 2011) and *in vivo* (Baker *et al.*, 2009, Peet & Baker, 2011). Similarly, 12-*epi*-(3) reportedly exhibits higher affinity than 12-*epi*-(1) (Béguin *et al.*, 2012). Further extension or branching of the terminal alkyl chain reduces affinity (Munro *et al.*, 2008). Thus, the ethoxymethyl substituent appears to confer optimal affinity and potency. Like (1), (3) is also metabolized more slowly than (2) (Hooker *et al.*, 2009). Based on the above hypothesis that the C22 methyl groups in (1) and (2) address different regions of the binding pocket, ethoxyethyl ether (4) was designed in that hope that it would interact with both of these regions, maximizing affinity. However, upon testing, (4) proved to have much lower affinity and potency than (3) (Munro *et al.*, 2008, Prevatt-Smith *et al.*, 2011). Indeed, all derivatives tested to date featuring substituted acetals, such as (5), exhibit reduced affinity and potency (Munro *et al.*, 2008, Prevatt-Smith *et al.*, 2011). These surprising and disappointing results cast doubt on the proposed binding model. We therefore determined the structure of (1) by single-crystal X-ray diffraction to obtain conformational information (Figure 2).

Other than the disordered furan ring, the neoclerodane scaffold is almost perfectly superimposable (r.m.s. < 0.1 Å) upon that of (2), as expected (Ortega *et al.*, 1982). However, the resulting relationship between the acetate and the MOM ether was unexpected. Both O3 and C22 in (1) overlap with their counterparts in (2), being separated by just 0.9 Å (O3) and 1.2 Å (C22) – less than their atomic radii. The overlapping van der Waals surfaces of O3 and C22 are shown in Figure 3. This result was surprising, given the different point of attachment of C22 in these two compounds (Figure 1). This counterintuitive result occurs because both bonds to the acetal carbon C21 in (1) are *gauche* (torsion angles: 69.8° (O2—C22) and 76.5° (C2—O3)), allowing the ether to trace a part helix around the planar acetate in (2). This is known as the `classic anomeric' conformation (Anderson, 2000, Brameld *et al.*, 2008). Generally, solid-state conformations coincide closely with the bioactive conformation of the protein-bound ligand (Brameld *et al.*, 2008). This is because both solid-state and bound conformations tend toward the free energy minimum. The similarity is greatest in high-affinity ligands, since any change in conformation during binding requires energy, and this `energetic penalty' reduces affinity (Brameld *et al.*, 2008). As discussed above, structure-activity studies indicate that O3 and C22 contribute substantially to binding of both (2) and (1). The near-superimposability of these atoms in the crystal structures of these two high-affinity ligands suggests that they may represent similar bioactive conformations. Alkoxymethyl ethers invariably adopt the classic anomeric conformation, due to strong anomeric interactions involving both O atoms (Anderson, 2000, Brameld *et al.*, 2008). Interestingly, however, substitution of the acetal carbon introduces steric interactions which greatly reduce this preference. With a methyl substituent, as in (4), the classic anomeric conformation predominates, but is not exclusive. With larger substituents this conformation is strongly disfavoured, and rarely occurs (Anderson, 2000). If the classic anomeric conformation seen in (1) is optimal for binding, acetal substitution would therefore be expected to reduce affinity by this conformational influence, even if the substituents do not themselves interact unfavourably with the receptor. This may contribute to the dramatic reductions in affinity and potency seen even with small acetal substituents (Munro *et al.*, 2008, Prevatt-Smith *et al.*, 2011).

The recently reported crystal structure of the tetrahydropyranyl (THP) ether (5) illustrates this point (Prevatt-Smith *et al.*, 2011). The cyclic acetal does not adopt the classic anomeric conformation, and superimposition on (2) gives much poorer overlap than seen with (1) (Figure 4). Acetal oxygen O3 is separated from its counterpart in (2) by 2.5 Å, and is instead almost coincident with C22 (<0.2 Å). Furthermore, the THP ring is disordered, consisting of a mixture of two interconvertible chair conformations. Thus, the THP ether exhibits weaker conformational preferences than the MOM ether, and much poorer overlap with (2). This may partly account for its lower potency. Our results suggest a possible conformational basis for the high binding affinity of salvinorin B alkoxymethyl ethers such as (1) and (3), and for the reduced affinity of substituted acetal derivatives such as (4) and (5). As a structurally atypical and extremely potent agonist, the structure of (1) reported here may prove useful in modelling the activation of the κ opioid receptor.

### Experimental

Compound (1) was prepared as described previously, by treatment of salvinorin B with CH_3_OCH_2_Cl and *i*-Pr_2_NEt in anhydrous CH_2_Cl_2_, and purified by flash chromatography on silica gel (Béguin *et al.*, 2009). Amended characterization data have been reported elsewhere (Munro *et al.*, 2008). Dissolution of 200 mg in minimal boiling methanol (~3 ml) and slow cooling gave colourless needles, mp 165–167 °C (438–440 K).

### Refinement

The 3-furyl substituent was treated with a two-site disorder model consisting of [O8, C13, C14, C15, C16] and [O8*, C13*, C14*, C15*, C16*] with refined site occupancy factors of 0.66 (2) and 0.34 (2), respectively. These ten atoms were included in the least-squares refinement with rigid bond, similar *U^ij^*, common plane and 1,2-distance restraints. All H atoms were allowed to ride on their respective C atoms with C—H distances constrained to the *SHELXTL* (Sheldrick, 2008) default values for the specified functional groups at 193 K, *i.e.*, 0.95, 0.98, 0.99 and 1.00 Å for the olefic, methyl, methylene and methine H atoms, respectively. The *U*_iso_(H) values were set at 1.5*U*_eq_(C) for the methyl H atoms and 1.2*U*_eq_(C) for all others. The Flack parameter obtained [*x* = 0.2 (7)] was inconclusive. The absolute stereochemistry of salvinorin B has been established *via* the *p*-bromobenzoate (Tidgewell *et al.*, 2006).

### Crystal data

**Table:**

C_23_H_30_O_8_	*F*(000) = 928
*M**_r_* = 434.47	*D*_x_ = 1.355 Mg m^−^^3^
Monoclinic, *C*2	Melting point = 438–440 K
Hall symbol: C 2y	Mo *K*α radiation, λ = 0.71073 Å
*a* = 27.8848 (7) Å	Cell parameters from 9071 reflections
*b* = 6.2415 (2) Å	θ = 3.0–29.5°
*c* = 12.8212 (3) Å	µ = 0.10 mm^−^^1^
β = 107.351 (1)°	*T* = 193 K
*V* = 2129.9 (1) Å^3^	Needle, colourless
*Z* = 4	0.25 × 0.13 × 0.07 mm

### Data collection

**Table:**

Bruker APEXII CCD diffractometer	6195 independent reflections
Radiation source: fine-focus sealed tube	5296 reflections with *I* > 2σ(*I*)
Graphite monochromator	*R*_int_ = 0.033
Detector resolution: 836.6 pixels mm^-1^	θ_max_ = 30.0°, θ_min_ = 1.9°
ω scans, 2580 0.5° rotations	*h* = −38→38
Absorption correction: multi-scan (*SADABS*; Bruker, 2004)	*k* = −8→8
*T*_min_ = 0.975, *T*_max_ = 0.993	*l* = −18→18
27784 measured reflections	

### Refinement

**Table:**

Refinement on *F*^2^	Primary atom site location: structure-invariant direct methods
Least-squares matrix: full	Secondary atom site location: difference Fourier map
*R*[*F*^2^ > 2σ(*F*^2^)] = 0.040	Hydrogen site location: inferred from neighbouring sites
*wR*(*F*^2^) = 0.108	H-atom parameters constrained
*S* = 1.04	*w* = 1/[σ^2^(*F*_o_^2^) + (0.062*P*)^2^ + 0.2734*P*] where *P* = (*F*_o_^2^ + 2*F*_c_^2^)/3
6195 reflections	(Δ/σ)_max_ = 0.001
330 parameters	Δρ_max_ = 0.28 e Å^−^^3^
181 restraints	Δρ_min_ = −0.16 e Å^−^^3^

### Special details

**Table:**

Refinement. Refinement of *F*^2^ against ALL reflections. The weighted *R*-factor *wR* and goodness of fit *S* are based on *F*^2^, conventional *R*-factors *R* are based on *F*, with *F* set to zero for negative *F*^2^. The threshold expression of *F*^2^ > 2σ(*F*^2^) is used only for calculating *R*-factors(gt) *etc*. and is not relevant to the choice of reflections for refinement. *R*-factors based on *F*^2^ are statistically about twice as large as those based on *F*, and *R*-factors based on ALL data will be even larger.

### Fractional atomic coordinates and isotropic or equivalent isotropic displacement parameters (Å^2^)

**Table:**

	*x*	*y*	*z*	*U*_iso_*/*U*_eq_	Occ. (<1)
O1	0.34600 (4)	1.0459 (2)	0.20598 (9)	0.0363 (3)	
O2	0.37399 (4)	1.1271 (2)	0.02768 (10)	0.0382 (3)	
O3	0.28986 (5)	1.1470 (3)	−0.07126 (12)	0.0533 (3)	
O4	0.50180 (4)	0.3453 (2)	0.13791 (9)	0.0347 (2)	
O5	0.53410 (4)	0.6752 (2)	0.15051 (11)	0.0434 (3)	
O6	0.31648 (4)	0.4502 (3)	0.48120 (9)	0.0436 (3)	
O7	0.39036 (5)	0.3347 (3)	0.57679 (9)	0.0458 (3)	
O8	0.1529 (2)	0.3841 (16)	0.3150 (5)	0.0598 (16)	0.66 (2)
O8*	0.1624 (5)	0.306 (3)	0.3130 (11)	0.065 (3)	0.34 (2)
C1	0.36787 (5)	0.9006 (2)	0.17700 (11)	0.0249 (3)	
C2	0.38017 (5)	0.9135 (3)	0.06823 (11)	0.0283 (3)	
H2	0.3558	0.8201	0.0142	0.034*	
C3	0.43320 (5)	0.8377 (3)	0.07636 (12)	0.0315 (3)	
H3A	0.4360	0.8163	0.0019	0.038*	
H3B	0.4576	0.9499	0.1126	0.038*	
C4	0.44644 (5)	0.6293 (3)	0.14044 (11)	0.0257 (3)	
H4	0.4222	0.5171	0.1007	0.031*	
C5	0.44100 (5)	0.6503 (2)	0.25776 (11)	0.0228 (2)	
C6	0.45549 (5)	0.4362 (3)	0.31852 (12)	0.0267 (3)	
H6A	0.4396	0.3176	0.2690	0.032*	
H6B	0.4924	0.4176	0.3379	0.032*	
C7	0.43961 (5)	0.4217 (3)	0.42203 (11)	0.0274 (3)	
H7A	0.4498	0.2813	0.4576	0.033*	
H7B	0.4565	0.5355	0.4738	0.033*	
C8	0.38318 (5)	0.4474 (2)	0.39397 (11)	0.0244 (3)	
H8	0.3678	0.3340	0.3391	0.029*	
C9	0.36365 (5)	0.6644 (2)	0.34072 (11)	0.0237 (2)	
C10	0.38330 (5)	0.6924 (2)	0.23975 (11)	0.0223 (2)	
H10	0.3658	0.5780	0.1877	0.027*	
C11	0.30621 (5)	0.6350 (3)	0.30016 (11)	0.0291 (3)	
H11A	0.2906	0.7691	0.2645	0.035*	
H11B	0.2980	0.5199	0.2445	0.035*	
C12	0.28354 (5)	0.5783 (3)	0.39194 (12)	0.0320 (3)	
H12	0.2766	0.7162	0.4245	0.038*	0.66 (2)
H12*	0.2744	0.7138	0.4231	0.038*	0.34 (2)
C13	0.2346 (2)	0.4602 (14)	0.3521 (11)	0.0399 (14)	0.66 (2)
C14	0.2245 (3)	0.2544 (13)	0.3026 (9)	0.0608 (16)	0.66 (2)
H14	0.2486	0.1629	0.2865	0.073*	0.66 (2)
C15	0.1763 (4)	0.2117 (11)	0.2826 (5)	0.0577 (16)	0.66 (2)
H15	0.1602	0.0830	0.2510	0.069*	0.66 (2)
C16	0.1899 (2)	0.5337 (15)	0.3570 (8)	0.0508 (17)	0.66 (2)
H16	0.1847	0.6694	0.3853	0.061*	0.66 (2)
C13*	0.2374 (3)	0.442 (2)	0.351 (2)	0.032 (2)	0.34 (2)
C14*	0.2354 (5)	0.241 (2)	0.2978 (16)	0.044 (2)	0.34 (2)
H14*	0.2634	0.1718	0.2848	0.052*	0.34 (2)
C15*	0.1891 (5)	0.165 (2)	0.2686 (15)	0.062 (3)	0.34 (2)
H15*	0.1769	0.0408	0.2264	0.075*	0.34 (2)
C16*	0.1910 (4)	0.481 (3)	0.3591 (14)	0.042 (2)	0.34 (2)
H16*	0.1802	0.6040	0.3898	0.051*	0.34 (2)
C17	0.36481 (6)	0.4082 (3)	0.49147 (12)	0.0321 (3)	
C18	0.49892 (5)	0.5576 (3)	0.14339 (12)	0.0295 (3)	
C19	0.47495 (5)	0.8301 (3)	0.32085 (12)	0.0293 (3)	
H19A	0.5088	0.8135	0.3138	0.044*	
H19B	0.4766	0.8225	0.3982	0.044*	
H19C	0.4612	0.9692	0.2909	0.044*	
C20	0.37823 (6)	0.8467 (3)	0.42559 (12)	0.0314 (3)	
H20A	0.3662	0.8125	0.4881	0.047*	
H20B	0.3629	0.9810	0.3921	0.047*	
H20C	0.4149	0.8623	0.4505	0.047*	
C21	0.33854 (7)	1.1533 (4)	−0.07497 (15)	0.0486 (5)	
H21A	0.3445	1.2925	−0.1060	0.058*	
H21B	0.3433	1.0389	−0.1244	0.058*	
C22	0.27678 (9)	1.3292 (5)	−0.0192 (2)	0.0654 (6)	
H22A	0.2403	1.3332	−0.0332	0.098*	
H22B	0.2877	1.4595	−0.0482	0.098*	
H22C	0.2933	1.3206	0.0597	0.098*	
C23	0.55100 (6)	0.2564 (3)	0.14724 (16)	0.0407 (4)	
H23A	0.5492	0.0996	0.1474	0.061*	
H23B	0.5751	0.3056	0.2155	0.061*	
H23C	0.5620	0.3039	0.0851	0.061*	

### Atomic displacement parameters (Å^2^)

**Table:**

	*U*^11^	*U*^22^	*U*^33^	*U*^12^	*U*^13^	*U*^23^
O1	0.0463 (6)	0.0288 (5)	0.0338 (6)	0.0112 (5)	0.0120 (5)	0.0024 (4)
O2	0.0313 (5)	0.0392 (6)	0.0420 (6)	0.0014 (5)	0.0073 (4)	0.0178 (5)
O3	0.0365 (6)	0.0615 (9)	0.0539 (8)	0.0058 (6)	0.0015 (5)	−0.0023 (7)
O4	0.0311 (5)	0.0329 (5)	0.0440 (6)	0.0031 (4)	0.0170 (5)	−0.0081 (5)
O5	0.0319 (5)	0.0406 (7)	0.0629 (8)	−0.0012 (5)	0.0221 (5)	0.0062 (6)
O6	0.0355 (6)	0.0683 (8)	0.0309 (6)	0.0052 (6)	0.0160 (5)	0.0144 (6)
O7	0.0433 (6)	0.0649 (8)	0.0291 (6)	0.0023 (6)	0.0105 (5)	0.0140 (6)
O8	0.0397 (17)	0.096 (4)	0.0512 (16)	−0.019 (2)	0.0254 (14)	−0.016 (2)
O8*	0.047 (4)	0.086 (7)	0.072 (5)	−0.019 (4)	0.032 (3)	0.007 (4)
C1	0.0239 (6)	0.0249 (6)	0.0237 (6)	−0.0013 (5)	0.0039 (5)	−0.0001 (5)
C2	0.0283 (6)	0.0299 (7)	0.0266 (6)	−0.0006 (6)	0.0081 (5)	0.0056 (6)
C3	0.0312 (7)	0.0364 (7)	0.0297 (7)	0.0035 (6)	0.0135 (6)	0.0075 (6)
C4	0.0252 (6)	0.0288 (7)	0.0250 (6)	−0.0006 (5)	0.0103 (5)	0.0000 (5)
C5	0.0239 (6)	0.0211 (6)	0.0239 (6)	−0.0001 (5)	0.0077 (5)	0.0000 (5)
C6	0.0291 (7)	0.0223 (6)	0.0301 (7)	0.0026 (5)	0.0109 (5)	0.0021 (5)
C7	0.0289 (6)	0.0265 (6)	0.0269 (6)	0.0026 (5)	0.0082 (5)	0.0051 (5)
C8	0.0284 (6)	0.0227 (6)	0.0228 (6)	−0.0021 (5)	0.0086 (5)	−0.0002 (5)
C9	0.0257 (6)	0.0239 (6)	0.0227 (6)	0.0010 (5)	0.0094 (5)	−0.0004 (5)
C10	0.0247 (6)	0.0215 (6)	0.0213 (6)	−0.0004 (5)	0.0078 (5)	−0.0006 (5)
C11	0.0267 (6)	0.0371 (7)	0.0254 (7)	0.0017 (6)	0.0107 (5)	0.0028 (6)
C12	0.0308 (7)	0.0376 (8)	0.0306 (7)	0.0020 (6)	0.0137 (6)	0.0021 (6)
C13	0.045 (3)	0.040 (2)	0.037 (3)	−0.006 (2)	0.015 (3)	0.007 (2)
C14	0.060 (3)	0.046 (2)	0.083 (3)	0.001 (3)	0.033 (3)	0.001 (2)
C15	0.064 (4)	0.057 (3)	0.063 (2)	−0.024 (3)	0.035 (2)	−0.015 (2)
C16	0.043 (2)	0.071 (4)	0.042 (3)	−0.007 (2)	0.0173 (18)	−0.008 (3)
C13*	0.022 (3)	0.043 (5)	0.037 (5)	0.005 (3)	0.018 (3)	0.003 (4)
C14*	0.035 (3)	0.031 (3)	0.070 (4)	−0.011 (3)	0.024 (3)	−0.017 (3)
C15*	0.046 (5)	0.049 (4)	0.098 (6)	−0.022 (4)	0.031 (4)	−0.010 (4)
C16*	0.034 (4)	0.067 (5)	0.038 (4)	0.007 (3)	0.030 (3)	0.012 (4)
C17	0.0345 (7)	0.0362 (8)	0.0271 (7)	−0.0030 (6)	0.0114 (6)	0.0015 (6)
C18	0.0281 (7)	0.0346 (7)	0.0285 (7)	0.0012 (6)	0.0126 (6)	0.0014 (6)
C19	0.0300 (7)	0.0259 (6)	0.0309 (7)	−0.0046 (6)	0.0073 (5)	−0.0023 (6)
C20	0.0402 (8)	0.0281 (7)	0.0273 (7)	−0.0001 (6)	0.0121 (6)	−0.0041 (6)
C21	0.0445 (9)	0.0660 (12)	0.0357 (9)	0.0152 (9)	0.0126 (7)	0.0210 (9)
C22	0.0532 (12)	0.0669 (14)	0.0752 (15)	0.0227 (11)	0.0176 (11)	0.0014 (13)
C23	0.0338 (8)	0.0450 (9)	0.0468 (10)	0.0088 (7)	0.0172 (7)	−0.0058 (7)

### Geometric parameters (Å, º)

**Table:**

O1—C1	1.2112 (17)	C9—C11	1.5404 (19)
O2—C21	1.400 (2)	C9—C20	1.543 (2)
O2—C2	1.4225 (18)	C9—C10	1.5586 (17)
O3—C21	1.373 (2)	C10—H10	1.0000
O3—C22	1.420 (3)	C11—C12	1.5340 (19)
O4—C18	1.3305 (19)	C11—H11A	0.9900
O4—C23	1.4513 (18)	C11—H11B	0.9900
O5—C18	1.2067 (19)	C12—C13*	1.500 (3)
O6—C17	1.3404 (18)	C12—C13	1.501 (2)
O6—C12	1.4715 (19)	C12—H12	1.0000
O7—C17	1.2040 (19)	C12—H12*	1.0000
O8—C16	1.377 (3)	C13—C16	1.346 (3)
O8—C15	1.385 (3)	C13—C14	1.423 (3)
O8*—C16*	1.378 (3)	C14—C15	1.320 (3)
O8*—C15*	1.380 (3)	C14—H14	0.9500
C1—C10	1.5214 (18)	C15—H15	0.9500
C1—C2	1.5344 (18)	C16—H16	0.9500
C2—C3	1.5263 (19)	C13*—C16*	1.349 (3)
C2—H2	1.0000	C13*—C14*	1.423 (3)
C3—C4	1.524 (2)	C14*—C15*	1.320 (3)
C3—H3A	0.9900	C14*—H14*	0.9500
C3—H3B	0.9900	C15*—H15*	0.9500
C4—C18	1.5198 (19)	C16*—H16*	0.9500
C4—C5	1.5617 (18)	C19—H19A	0.9800
C4—H4	1.0000	C19—H19B	0.9800
C5—C19	1.5340 (19)	C19—H19C	0.9800
C5—C6	1.5390 (19)	C20—H20A	0.9800
C5—C10	1.5778 (17)	C20—H20B	0.9800
C6—C7	1.5216 (19)	C20—H20C	0.9800
C6—H6A	0.9900	C21—H21A	0.9900
C6—H6B	0.9900	C21—H21B	0.9900
C7—C8	1.5147 (19)	C22—H22A	0.9800
C7—H7A	0.9900	C22—H22B	0.9800
C7—H7B	0.9900	C22—H22C	0.9800
C8—C17	1.5054 (18)	C23—H23A	0.9800
C8—C9	1.5415 (19)	C23—H23B	0.9800
C8—H8	1.0000	C23—H23C	0.9800
			
C21—O2—C2	115.33 (14)	O6—C12—C13	106.9 (5)
C21—O3—C22	112.85 (17)	O6—C12—C11	114.68 (11)
C18—O4—C23	116.56 (13)	C13*—C12—C11	111.9 (9)
C17—O6—C12	123.97 (11)	C13—C12—C11	113.2 (5)
C16—O8—C15	106.2 (4)	O6—C12—H12	107.2
C16*—O8*—C15*	111.6 (8)	C13—C12—H12	107.2
O1—C1—C10	124.47 (12)	C11—C12—H12	107.2
O1—C1—C2	120.59 (12)	O6—C12—H12*	108.8
C10—C1—C2	114.89 (11)	C13*—C12—H12*	108.8
O2—C2—C3	108.95 (11)	C11—C12—H12*	108.8
O2—C2—C1	110.23 (12)	C16—C13—C14	105.5 (4)
C3—C2—C1	113.35 (11)	C16—C13—C12	125.2 (5)
O2—C2—H2	108.1	C14—C13—C12	129.3 (6)
C3—C2—H2	108.1	C15—C14—C13	108.8 (4)
C1—C2—H2	108.1	C15—C14—H14	125.6
C4—C3—C2	112.05 (11)	C13—C14—H14	125.6
C4—C3—H3A	109.2	C14—C15—O8	109.2 (4)
C2—C3—H3A	109.2	C14—C15—H15	125.4
C4—C3—H3B	109.2	O8—C15—H15	125.4
C2—C3—H3B	109.2	C13—C16—O8	110.3 (4)
H3A—C3—H3B	107.9	C13—C16—H16	124.8
C18—C4—C3	109.94 (11)	O8—C16—H16	124.8
C18—C4—C5	111.73 (11)	C16*—C13*—C14*	107.1 (6)
C3—C4—C5	111.70 (11)	C16*—C13*—C12	128.0 (8)
C18—C4—H4	107.8	C14*—C13*—C12	124.9 (8)
C3—C4—H4	107.8	C15*—C14*—C13*	110.3 (8)
C5—C4—H4	107.8	C15*—C14*—H14*	124.8
C19—C5—C6	109.92 (11)	C13*—C14*—H14*	124.8
C19—C5—C4	110.34 (10)	C14*—C15*—O8*	104.9 (9)
C6—C5—C4	109.16 (11)	C14*—C15*—H15*	127.6
C19—C5—C10	113.43 (11)	O8*—C15*—H15*	127.6
C6—C5—C10	108.71 (10)	C13*—C16*—O8*	105.6 (7)
C4—C5—C10	105.12 (10)	C13*—C16*—H16*	127.2
C7—C6—C5	113.11 (11)	O8*—C16*—H16*	127.2
C7—C6—H6A	109.0	O7—C17—O6	117.96 (13)
C5—C6—H6A	109.0	O7—C17—C8	124.09 (14)
C7—C6—H6B	109.0	O6—C17—C8	117.89 (12)
C5—C6—H6B	109.0	O5—C18—O4	123.31 (13)
H6A—C6—H6B	107.8	O5—C18—C4	125.31 (14)
C8—C7—C6	109.78 (11)	O4—C18—C4	111.38 (12)
C8—C7—H7A	109.7	C5—C19—H19A	109.5
C6—C7—H7A	109.7	C5—C19—H19B	109.5
C8—C7—H7B	109.7	H19A—C19—H19B	109.5
C6—C7—H7B	109.7	C5—C19—H19C	109.5
H7A—C7—H7B	108.2	H19A—C19—H19C	109.5
C17—C8—C7	111.86 (11)	H19B—C19—H19C	109.5
C17—C8—C9	110.35 (11)	C9—C20—H20A	109.5
C7—C8—C9	113.75 (11)	C9—C20—H20B	109.5
C17—C8—H8	106.8	H20A—C20—H20B	109.5
C7—C8—H8	106.8	C9—C20—H20C	109.5
C9—C8—H8	106.8	H20A—C20—H20C	109.5
C11—C9—C8	103.83 (11)	H20B—C20—H20C	109.5
C11—C9—C20	110.78 (11)	O3—C21—O2	113.10 (14)
C8—C9—C20	110.59 (11)	O3—C21—H21A	109.0
C11—C9—C10	108.70 (10)	O2—C21—H21A	109.0
C8—C9—C10	107.52 (10)	O3—C21—H21B	109.0
C20—C9—C10	114.80 (11)	O2—C21—H21B	109.0
C1—C10—C9	114.89 (11)	H21A—C21—H21B	107.8
C1—C10—C5	109.55 (10)	O3—C22—H22A	109.5
C9—C10—C5	117.10 (10)	O3—C22—H22B	109.5
C1—C10—H10	104.6	H22A—C22—H22B	109.5
C9—C10—H10	104.6	O3—C22—H22C	109.5
C5—C10—H10	104.6	H22A—C22—H22C	109.5
C12—C11—C9	113.17 (11)	H22B—C22—H22C	109.5
C12—C11—H11A	108.9	O4—C23—H23A	109.5
C9—C11—H11A	108.9	O4—C23—H23B	109.5
C12—C11—H11B	108.9	H23A—C23—H23B	109.5
C9—C11—H11B	108.9	O4—C23—H23C	109.5
H11A—C11—H11B	107.8	H23A—C23—H23C	109.5
O6—C12—C13*	103.5 (10)	H23B—C23—H23C	109.5
			
C21—O2—C2—C3	114.88 (14)	C20—C9—C11—C12	−60.11 (16)
C21—O2—C2—C1	−120.16 (14)	C10—C9—C11—C12	172.88 (12)
O1—C1—C2—O2	14.32 (18)	C17—O6—C12—C13*	130.8 (6)
C10—C1—C2—O2	−167.98 (11)	C17—O6—C12—C13	134.9 (4)
O1—C1—C2—C3	136.74 (15)	C17—O6—C12—C11	8.6 (2)
C10—C1—C2—C3	−45.56 (16)	C9—C11—C12—O6	−32.17 (18)
O2—C2—C3—C4	168.32 (12)	C9—C11—C12—C13*	−149.7 (8)
C1—C2—C3—C4	45.19 (17)	C9—C11—C12—C13	−155.2 (4)
C2—C3—C4—C18	178.41 (12)	O6—C12—C13—C16	116.8 (10)
C2—C3—C4—C5	−56.95 (16)	C11—C12—C13—C16	−115.9 (10)
C18—C4—C5—C19	65.23 (14)	O6—C12—C13—C14	−63.2 (12)
C3—C4—C5—C19	−58.40 (14)	C11—C12—C13—C14	64.0 (12)
C18—C4—C5—C6	−55.66 (14)	C16—C13—C14—C15	−1.4 (9)
C3—C4—C5—C6	−179.29 (11)	C12—C13—C14—C15	178.7 (12)
C18—C4—C5—C10	−172.13 (12)	C13—C14—C15—O8	1.2 (9)
C3—C4—C5—C10	64.24 (13)	C16—O8—C15—C14	−0.6 (8)
C19—C5—C6—C7	72.79 (14)	C14—C13—C16—O8	1.0 (9)
C4—C5—C6—C7	−166.06 (11)	C12—C13—C16—O8	−179.0 (11)
C10—C5—C6—C7	−51.90 (14)	C15—O8—C16—C13	−0.3 (9)
C5—C6—C7—C8	59.29 (15)	O6—C12—C13*—C16*	112 (2)
C6—C7—C8—C17	173.22 (12)	C11—C12—C13*—C16*	−123.9 (19)
C6—C7—C8—C9	−60.92 (15)	O6—C12—C13*—C14*	−67.1 (19)
C17—C8—C9—C11	−63.99 (13)	C11—C12—C13*—C14*	57 (2)
C7—C8—C9—C11	169.34 (11)	C16*—C13*—C14*—C15*	3.1 (17)
C17—C8—C9—C20	54.87 (14)	C12—C13*—C14*—C15*	−178 (2)
C7—C8—C9—C20	−71.79 (14)	C13*—C14*—C15*—O8*	−5.8 (16)
C17—C8—C9—C10	−179.08 (11)	C16*—O8*—C15*—C14*	6.7 (16)
C7—C8—C9—C10	54.26 (14)	C14*—C13*—C16*—O8*	1.1 (16)
O1—C1—C10—C9	6.55 (19)	C12—C13*—C16*—O8*	−178 (2)
C2—C1—C10—C9	−171.04 (11)	C15*—O8*—C16*—C13*	−4.9 (17)
O1—C1—C10—C5	−127.68 (14)	C12—O6—C17—O7	167.27 (17)
C2—C1—C10—C5	54.72 (14)	C12—O6—C17—C8	−15.2 (2)
C11—C9—C10—C1	68.63 (14)	C7—C8—C17—O7	−10.3 (2)
C8—C9—C10—C1	−179.56 (11)	C9—C8—C17—O7	−138.04 (17)
C20—C9—C10—C1	−56.05 (15)	C7—C8—C17—O6	172.30 (14)
C11—C9—C10—C5	−160.70 (11)	C9—C8—C17—O6	44.60 (18)
C8—C9—C10—C5	−48.89 (14)	C23—O4—C18—O5	3.4 (2)
C20—C9—C10—C5	74.63 (15)	C23—O4—C18—C4	−176.09 (12)
C19—C5—C10—C1	58.93 (13)	C3—C4—C18—O5	36.7 (2)
C6—C5—C10—C1	−178.48 (11)	C5—C4—C18—O5	−87.90 (17)
C4—C5—C10—C1	−61.70 (13)	C3—C4—C18—O4	−143.75 (13)
C19—C5—C10—C9	−74.18 (14)	C5—C4—C18—O4	91.63 (15)
C6—C5—C10—C9	48.42 (15)	C22—O3—C21—O2	69.8 (2)
C4—C5—C10—C9	165.19 (11)	C2—O2—C21—O3	76.5 (2)
C8—C9—C11—C12	58.63 (15)		
